# Soil and climate factors affect the nutrient resorption characteristics of desert shrub roots in Xinjiang, China

**DOI:** 10.3389/fpls.2025.1518846

**Published:** 2025-06-27

**Authors:** Yan Luo, Wenya Wei, Yaxuan Wang, Tianai Xue, Kaijuan Du

**Affiliations:** ^1^ College of Ecology and Environment, Xinjiang University, Urumqi, China; ^2^ Key Laboratory of Oasis Ecology, Ministry of Education, Xinjiang University, Urumqi, China; ^3^ Xinjiang Jinghe Observation and Research Station of Temperate Desert Ecosystem, Ministry of Education, Xinjiang University, Urumqi, China

**Keywords:** desert shrub, root, nutrient resorption efficiency, nutrient limitation, environmental factors

## Abstract

**Introduction:**

Nutrient resorption is a vital nutrient utilization strategy in desert plants and is essential for understanding desert ecosystems and addressing climate change. Although the resorption characteristics in plants have been studied extensively, those of desert plant roots remain insufficiently explored.

**Methods:**

This study investigated the concentrations of nitrogen, phosphorus, and potassium, as well as their resorption efficiencies, in 21 shrubs within a desert ecosystem in Xinjiang, Northwest China. Our study was designed to compare nutrient resorption efficiency patterns among shrub species and assess how the these patterns respond to variations in climatic conditions and edaphic properties.

**Results:**

The results indicated that nitrogen resorption efficiency (NRE), phosphorus resorption efficiency (PRE), and potassium resorption efficiency (KRE) for all plants were 29.14 ± 0.98%, 37.58 ± 0.92%, and 42.20 ± 0.93%, respectively. Among functional groups, angiosperms exhibited higher PRE (36.31 ± 1.00%) and KRE (41.85 ± 0.98%) than gymnosperms. C_4_ plants (44.88 ± 1.53%) had significantly higher KRE than C_3_ plants (40.85 ± 1.17%). Among different families, *Tamaricaceae* had significantly higher NRE (33.84 ± 2.07%) and PRE (46.23 ± 1.72%) compared to others, while *Solanaceae* had the lowest KRE (33.84 ± 2.07%). Plant nutrient resorption efficiency is regulated by multiple environmental factors. Specifically, soil total phosphorus (STP) and total potassium (STK) serve as the primary drivers of NRE, while electrical conductivity (EC) and aridity index (AI) play critical roles in modulating PRE. Climate factors exhibit distinct influences: AI shows positive correlations with PRE in C_3 _plants and with NRE in C4 plants. MAT negatively affects KRE in C4 plants, whereas MAP exerts a positive effect on it. Notably, *Polygonaceae* plants demonstrate unique response patterns: NRE is jointly regulated by MAP and MAT, PRE is predominantly influenced by MAT and AI, and KRE depends on the combined influence of MAP and AI.

**Discussion:**

Our research further explores the mechanisms of nutrient cycling in desert ecosystems by analyzing the root nutrient resorption strategies of desert plants. This provides theoretical support for understanding how plants in desert ecosystems efficiently utilize limited nutrient resources under extreme drought conditions.

## 1 Introduction

Nitrogen (N), phosphorus (P), and potassium (K) are three essential nutrients for plant growth and play crucial roles in various physiological functions (Chapin, 1980; Elser et al., 2007; Du et al., 2020; Lopez et al., 2023). N and P are typically regarded as the primary limiting factors in terrestrial ecosystems (Sardans et al., 2014; Liao et al., 2024), while K not only plays a crucial role in key processes such as photosynthesis, water regulation, and stress resistance, but it is also vital for plant growth and development. Especially in arid ecosystems, the demand for K in plants is actually higher than for P (Sardans and Peñuelas, 2015; Jungová et al., 2023; Das and Mondal, 2024). When nutrient supplies are limited, plants have evolved nutrient resorption strategies to enhance nutrient use efficiency and reduce reliance on external sources. This process involves resorbing nutrients from senesced tissues and transferring them to green tissues, thereby enhancing their adaptation to nutrient-poor environments (Killingbeck, 1996; Reed et al., 2012; Brant and Chen, 2015; Zhang J. et al., 2024). Nutrient resorption efficiency (NuRE), as a measure of nutrient resorption, is defined as the nutrient difference between mature and senescent leaves and the ratio of nutrients in mature leaves (Reich and Oleksyn, 2004). Desert ecosystems exhibit extremely low soil nutrient concentrations, prompting plants to depend on internal recycling and resorption to adapt to nutrient-poor conditions (Lambers, 2022; Tariq et al., 2024; Gao et al., 2025). By maximizing nutrient reclamation, these plants can enhance their utilization efficiency. To gain a deeper understanding of nutrient cycling and resource-use strategies, studying nutrient resorption in desert areas is essential.

Nutrient resorption is a vital strategy used by desert plants to thrive in arid, low-nutrient environments (Drenovsky and Richards, 2006; Reichert et al., 2022; Zhao et al., 2024). Desert plants mitigate nutrient limitation by improving nutrient resorption efficiency. For instance, shrub leaves in the Chihuahua Desert exhibit significantly higher NRE and PRE compared to other regions (Killingbeck, 1993). However, studies suggest that the nitrogen resorption efficiency (NRE) and phosphorus resorption efficiency (PRE) of shrub leaves in the Chihuahua and Gurbantunggut deserts are not significantly higher than those in other areas (Killingbeck and Whitford, 2001; Zhang et al., 2018). Additionally, studies have shown that the PRE in desert plant leaves is generally higher than the NRE. This phenomenon is consistent with the relative resorption hypothesis, which posits that plants tend to preferentially absorb limiting nutrients (Han et al., 2013). K is of crucial significance, under drought-stress conditions, it maintains ion homeostasis and regulates osmotic balance, enabling plants to better adapt to water deficits, thereby enhancing their survival and ecological adaptability (Lebaudy et al., 2007; Sardans and Peñuelas, 2015; Gupta et al., 2020; Johnson et al., 2022). The potassium resorption efficiency (KRE) of leaves in the Xinjiang Desert is lower than that of global woody plants, likely due to soil nutrient scarcity and the adaptation strategies of the plants (Luo et al., 2020). Previous studies on plant nutrient resorption have predominantly focused on leaf tissues, with limited research on nutrient resorption characteristics of roots (Zheng et al., 2018). Roots are vital structures for water and nutrient absorption in plants and play crucial roles in nutrient acquisition and storage (Bardgett et al., 2014; Weemstra et al., 2016; Freschet and Roumet, 2017; Liu et al., 2022, 2024). Some studies have reported minimal or no significant changes in nutrient concentrations between live and dead roots (Aerts, 1996). Gordon and Jackson (2000) emphasized that roots function as sites for nutrient accumulation and as potential sources of nutrients. Desert plants enhance their ability to absorb limited water and nutrients by increasing root hair density and length and accumulating osmoregulatory substances, which helps maintain cellular osmotic pressure, prevents dehydration, and ensures normal nutrient resorption (Freschet et al., 2010; Gao et al., 2024; Wang et al., 2025). Additionally, they often form symbiotic relationships with arbuscular mycorrhizal fungi, significantly improving their efficiency in absorbing soil nutrients, especially phosphorus (Tariq et al., 2024). However, the extent to which roots contribute to nutrient resorption remains inadequately documented, primarily because of challenges in collection methods and associated costs. Further investigation into root nutrient resorption characteristics will deepen our understanding of plant resource utilization strategies in nutrient-poor environments and provide insights into their performance and ecological adaptation mechanisms.

The climate, soil nutrients, and plant characteristics are among the key driving forces of nutrient resorption (Reed et al., 2012; Yan et al., 2018; Huang et al., 2018). Climate, soil nutrients, and plant characteristics drive nutrient resorption (She et al., 2024; Wang et al., 2025). Key climatic factors include temperature, precipitation, and the aridity index. Suitable temperatures boost enzyme and transporter activities, enhancing nutrient absorption and transport, and increase fine root biomass and absorption area (Zhang S. et al., 2024). Precipitation enhances soil moisture, aeration, and microbial activity, promoting nutrient availability and root zone access. Under drought, plants adapt by increasing root hairs and accumulating osmoregulatory substances to maintain osmotic pressure and prevent dehydration (Li et al., 2025a). High temperatures in deserts can both enhance and inhibit enzyme activity, depending on thresholds, and induce stress hormones like ABA, affecting nutrient uptake and distribution (Beugnon et al., 2024). Plants also adapt to water stress by adjusting root architecture, leaf morphology, and physiological metabolism (Li et al., 2025b). Studies indicate that plants adapt to water stress through various strategies, including adjusting root architecture to increase water uptake capacity, changing leaf morphology to reduce transpiration, and regulating physiological metabolic processes (Delgado-Baquerizo et al., 2013; Williams and de Vries, 2020). These response mechanisms help plants to more efficient nutrient acquisition and utilization in nutrient-poor environments. Resorption efficiency may decrease due to drought (Zhang et al., 2018). However, some studies have indicated that drought does not inhibit resorption but rather plays a positive role in this process (Lobo-do-Vale et al., 2019; Luo et al., 2024). Soil nutrients directly influence resorption efficiency and are fundamental for plant growth (He et al., 2015). Studies have demonstrated that reduced soil moisture in arid regions adversely affects the resorption capacity of plants. This limitation occurs primarily because the soil nutrient diffusion is restricted. Moreover, the activities of soil and root enzymes decrease simultaneously, contributing to a nutrient-limiting ecological state (Sardans et al., 2017; Li et al., 2022). In arid regions, soil salinity is a major limiting factor for plant growth (James et al., 2005; Zhang S. et al., 2024). Conversely, some studies have shown that soil salinity has a minimal effect on the nutrient resorption of desert plant leaves (Wang L. et al., 2020). Desert plants employ various strategies to manage drought stress and the efficiency of nutrient resorption varies among species. Previous studies have demonstrated that the NRE of *Lycium ruthenicum* surpasses that of other elements, whereas the PRE of *Halostachys caspica* is greater than that of other shrubs in desert environments (Luo et al., 2020). Angiosperms and gymnosperms differ in their adaptations to high temperatures and drought stress. Angiosperms have complex roots and metabolic regulation, enhancing nutrient absorption through root hair density and organic acid secretion (Langguth et al., 2024). In contrast, gymnosperms have drought-resistant features like thick cuticles and low stomatal density, reducing water loss. Under prolonged drought, they optimize stomatal closure and root growth via ABA signaling (Kim et al., 2024; Rizzuto et al., 2024). Investigating the nutrient resorption mechanisms of desert plants and their responses to environmental changes will elucidate how these plants adapt to extreme conditions and provide a scientific basis for the restoration and management of desert ecosystems.

Desert plants are able to adapt to poor environments for a long time, so they have developed unique nutrient utilization strategies including optimized root architecture, efficient nutrient recovery mechanisms, and special photosynthesis mechanisms. Although the resorption of nutrients from the leaves has been extensively studied, the mechanisms underlying root nutrient resorption remain unclear. Xinjiang is the most widely distributed desert province in China, where shrubs occupy a significant ecological niche. In this study, we selected 21 shrub species from the Xinjiang desert region to analyze the concentration of N, P, and K in their roots. Aims to answer the following scientific questions: (1) What are the N, P and K stoichiometric characteristics of desert shrub plant roots and what is the relationship between these characteristic and nutrient resorption? (2) how do climate and soil factors affect the nutrient resorption characteristics of desert shrub roots? Our study aimed to elucidate nutrient utilization strategies for shrub roots in arid desert ecosystems. Understanding how desert shrubs optimize resorption of essential nutrients will improve our knowledge of plant adaptation to extreme environments.

## 2 Materials and methods

### 2.1 Study area

**Figure 1:**
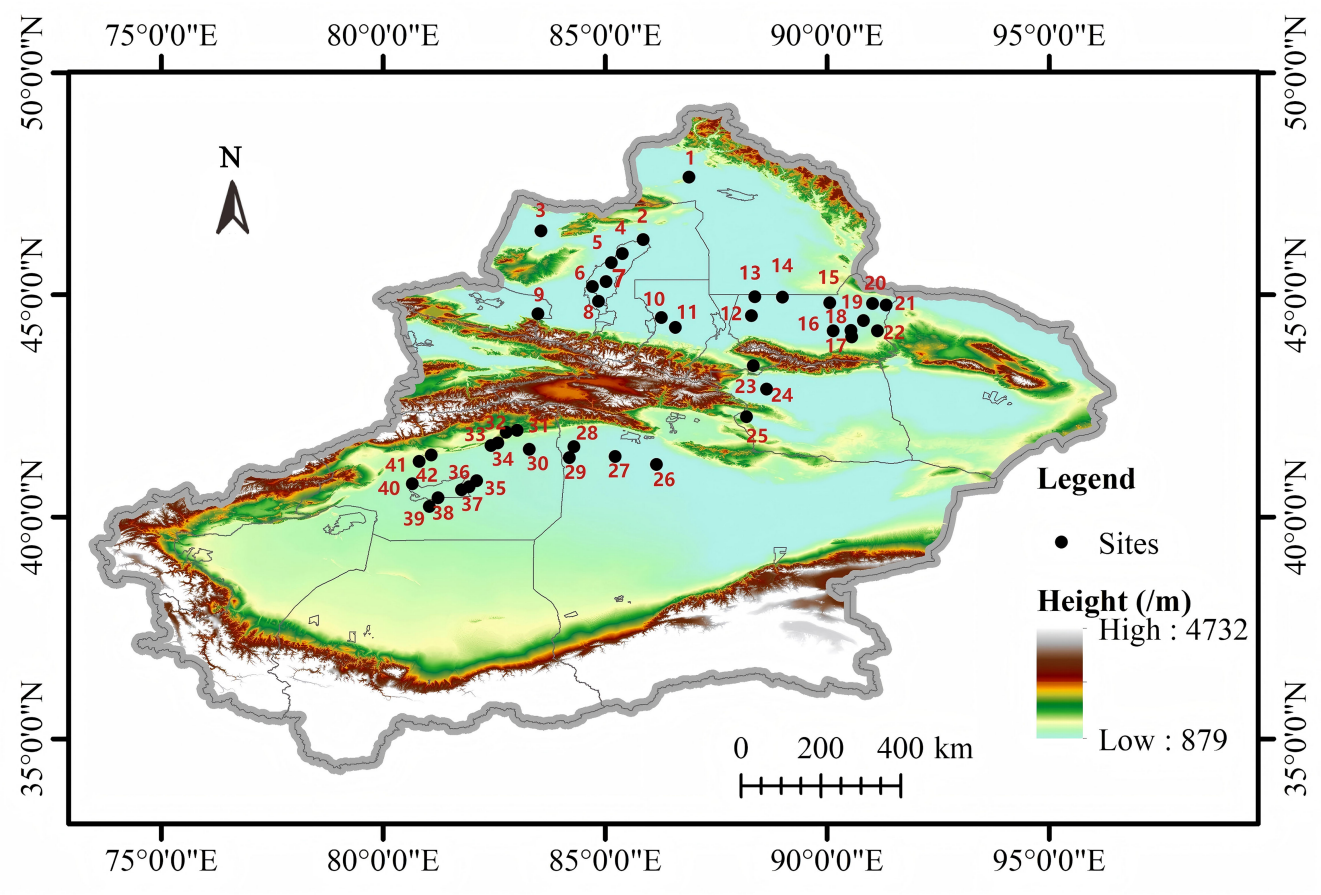
Overview of the study area.

This study was conducted across 42 field sites located in the desert region of Xinjiang (38.86-48.19°N, 77.06-93.44°E), with altitudes ranging from 270 to 1451 m (**Figure 1**; **Supplementary Table S1**). The study area is characterized by a temperate, continental arid climate featuring high temperatures and drought conditions during the summer months and low temperatures in winter. The mean annual temperature (MAT) ranged from 5.29 to 14.35 °C, while the mean annual precipitation (MAP) varied from 38 to 226 mm. The precipitation levels were higher during the summer months and lower in the winter, exhibiting a significant disparity in distribution throughout the year. The shrub species in the area include plants such as *Lycium ruthenicum*, *Nitraria tangutorum*, *Tamarix ramosissima*, and *Reaumuria soongorica* (**Supplementary Table S2**). According to the soil classification system established by the United States Department of Agriculture, the predominant soil types in the region are grey desert soil, gray-brown desert soil, and aeolian sandy soil. Soil salt concentration was assessed by electrical conductivity (EC), and the soil salt concentration in the study area was relatively high, as shown in **Supplementary Tables S3**, **S4**.

### 2.2 Sampling and measurement

We collected plant samples in mid-to-late July and mid-September in 2021. These specific sampling time periods were selected based on the phenological characteristics of desert plants in Xinjiang. In mid-to-late July, plants in Xinjiang desert areas are usually in their peak growth period. By mid-September, many desert plants in Xinjiang began to show signs of decline. Our study was conducted at 42 sampling sites in the desert region of Xinjiang. At each sampling point, we set a 20×20m quadrat. In each plot, 3 to 5 dominan plants with vigorous growth and consistent morphology were randomly selected, and 3–5 replicate icates were performed for each plant to ensure sample representativeness and reliability of ecological data. Coarse roots with diameters greater than 2 mm and a depth of approximately 50 cm below the surface were excavated. Soil particles and other substances were removed from the root surfaces. The roots were carefully rinsed with deionized water to remove any adhering soil particles and debris. Dried at 105 °C for 30 min and subsequently dried at 65 °C until a constant weight was achieved. They were then ground and stored for chemical analysis. Five soil samples at a depth of 0–50 cm were collected from each block using a soil sampler (XDB-Y, Jiangsu Xindacheng Instrument Co., Ltd, China.), and these samples were pooled together and fully mixed to remove organic debris and stones. Then a “four section” was used to spread the pooled soil into a thin layer, divided into four equal parts, and the four samples were recombined and mixed again. After screening, the samples were stored separately, transported to the laboratory, and spread the soil samples on a clean, non-reactive surface and placed them in a well-ventilated, cool, and shaded environment to ensure that the samples were protected from direct sunlight, dust, and chemical contamination. The dried soil was ground and then stored for chemical analysis.

At each sampling point, we collected 3–5 replicates based on the actual distribution. For the statistical analysis of the original dataset, we collected a total of 55 plants, 3 replicates, 58 plants, and 5 replicates for a total of 455 plant samples. The N concentration was determined using an Alpkem autoanalyzer (Kjektec System 1026 distillation unit, Sweden). Plant and soil samples were dried and ground to ensure uniformity. Subsequently, the N in the sample was distilled using the Kjeldahl method. After sulfuric acid digestion, the N was released as ammonia, and its concentration was determined by acid titration. The P content was determined using the molybdate/ascorbate blue colorimetric method with a ICP-OES instrument (7300 DV, PerkinElmer, United States). After chemical treatment, molybdate was added under specific pH conditions to generate a phosphorus-molybdenum blue complex. Using ascorbic acid as a reducing agent, this complex was further reduced to a blue compound, which was quantified by colorimetry. The method for determining potassium (K) concentration is described as follows: Accurately weigh 0.5 g of plant powder sample and place it into a polytetrafluoroethylene (PTFE) digestion vessel. Add 10 mL of concentrated nitric acid (HNO_3_, 65%) and 3 mL of hydrogen peroxide (H_2_O_2_, 30%). Seal the digestion vessel and place it into the microwave digestion system (Mars Xpress, CEM Corporation, USA) for digestion. After digestion, the sample is analyzed using inductively coupled plasma-optical emission spectrometry (ICP-OES). The instrument used is the Optima 7000 DV (PerkinElmer Inc., USA). The sample solution is nebulized and introduced into the plasma torch, where it is ionized into a plasma state at high temperature. Potassium emits characteristic spectra at a specific wavelength (766.49 nm), and its concentration is quantitatively determined by measuring the emission intensity (Ebrahim et al., 2014).

### 2.3 Meteorological data acquisition

MAT (°C) and MAP (mm) data were derived from WorldClim version 2.0 (http://worldclim.org/version2), and the drought index (AI) was derived from the meteorological database of the International Centre for Agricultural Research Spatial Information Consortium (http://www.cgiar-csi.org).

### 2.4 Nutrient resorption efficiency

Nutrient resorption efficiency (NuRE) was calculated as described by Vergutz et al. (2012):


(1) $$NuRE=\frac{Nu_{ma\text{tu}re}-Nu_{senesced}}{Nu_{mature}}\times 100\%$$

where, NuRE is the nutrient resorption efficiency, Nu_mature_ is the nutrient concentration in the roots during summer, and Nu_senesced_ is the nutrient concentration in the roots during autumn.

Relative nutrient resorption (RNuR) is calculated as described by Han et al. (2013):


(2) RNuR1=NRE−PRE

(3) RNuR2=NRE−KRE

(4) RNuR3=KRE−PRE

where, NRE, PRE, and KRE are the nutrient resorption efficiencies of N, P, and K, respectively.

### 2.5 Statistical analyses

**Figure 2:**
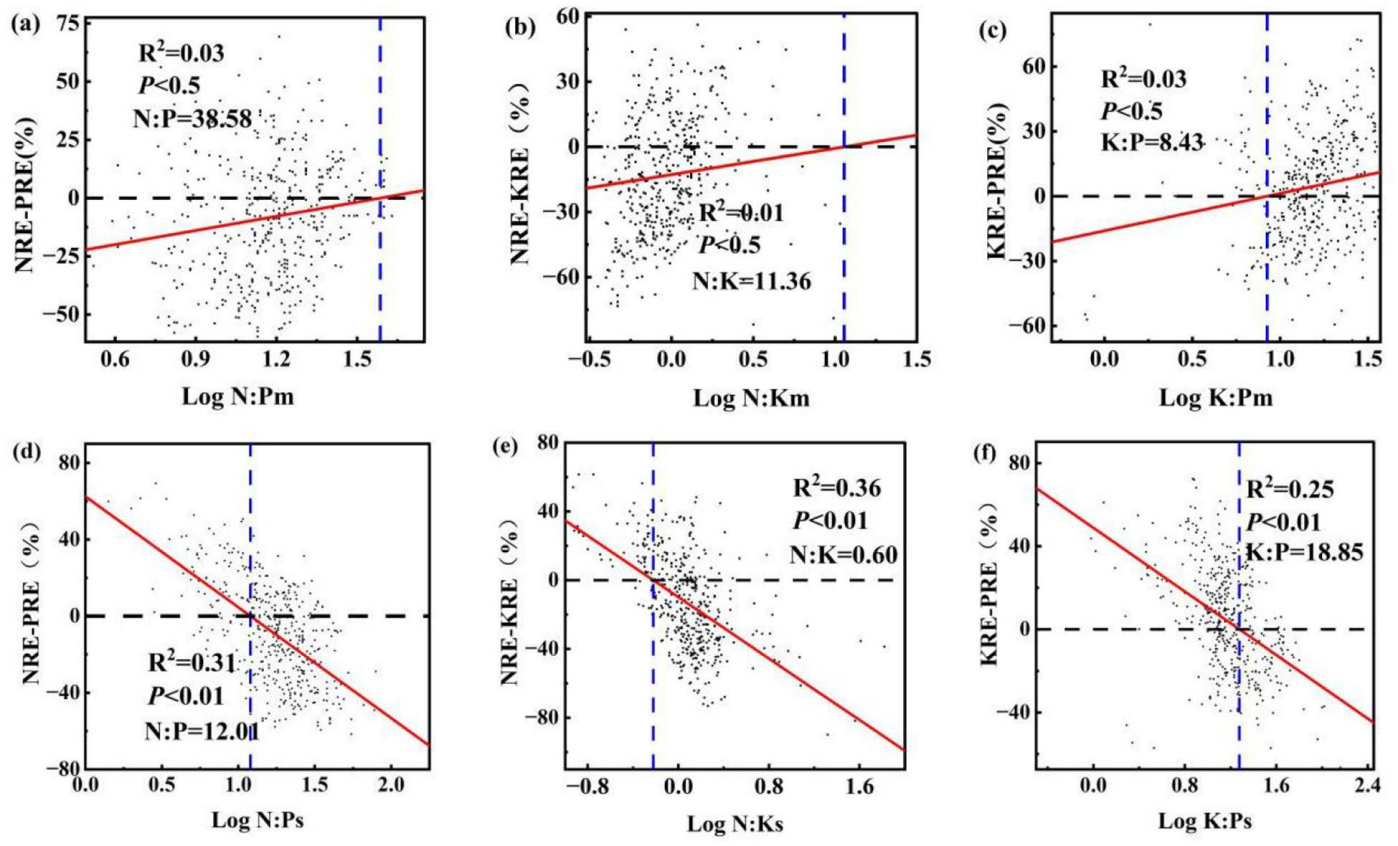
Ratios of N, P, K and relative resorption efficiency in summer and autumn roots Note: **(a)** N: P_m_ and NRE-KRE; **(b)** N: K_m_ and NRE-KRE; **(c)** K: P_m_ and KRE-PRE; **(d)** N: P_s_ and NRE-KRE; **(e)** N: K_s_ and NRE-KRE; **(f)** K: P_s_ and KRE-PRE. Horizontal black dashed line indicates the NRE equal to the critical N: P ratio of the PRE. The vertical blue dashed line crosses the black dashed line and the intersection of the red fitted curve is the corresponding ratio.

One-way analysis of variance was employed to determine the differences in N, P, and K stoichiometry and nutrient resorption efficiency among the course roots of the sampled individuals. Tukey’s HSD test was used to assess significant differences between multiple groups (*p<* 0.05). The relative nutrient resorption proportion between N and P was quantified as the difference between NRE and PRE, i.e., NRE-PRE. The stoichiometric ratio N:P was used to indicate the relative limitation of N versus P in plants. To identify critical thresholds, we performed regression analysis between the relative resorption efficiency (NRE-PRE) and the N:P ratio (log-transformed), with the results visualized in **Figure 2**. In the figure: The horizontal dashed line marks the equilibrium point where NRE equals PRE (NRE-PRE=0). The vertical dashed line indicates the critical N:P ratio derived from the regression model after logarithmic transformation (Han et al., 2013).

The impact of environmental variables on NRE, PRE, and KRE was investigated using a linear mixed model (LMM). Regression analysis of environmental factors was performed using the restricted maximum likelihood estimation method, with the model constructed using the nlme package in R version 4.3.3. The LMM was designed using NRE, PRE, and KRE as response variables. The analysis incorporated various plant types as random effects and climatic (MAT, MAP, and AI) and soil factors (STN, STP, STK, pH, EC, and K_soil_) as fixed effects. The RDA analysis of climate and nutrient reabsorption efficiency was performed using the “vegan” package in R. All data processing, analyses, and visualizations were performed using Microsoft Excel 2013, Origin 2024, and R version 4.3.3.

## 3 Results

### 3.1 Stoichiometric characteristics of N, P and K in roots of desert shrubs

**Figure 3:**
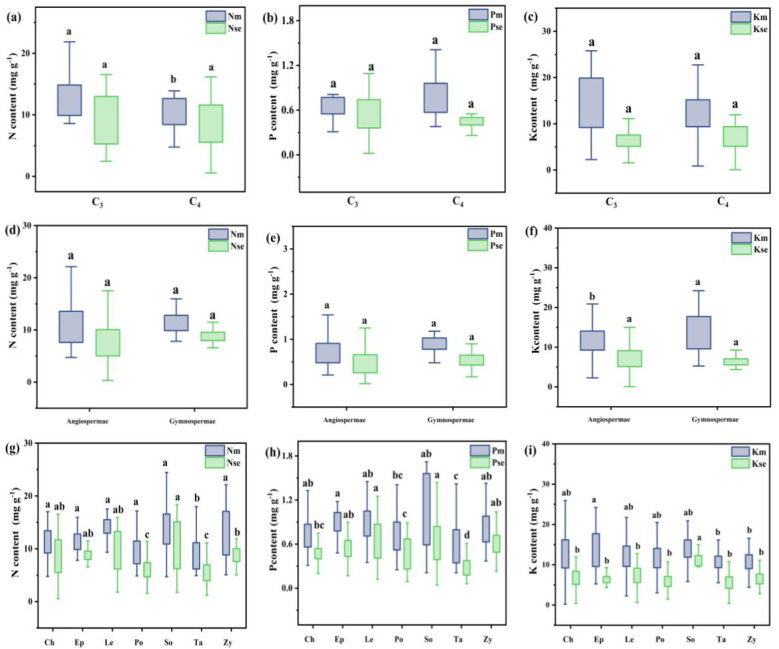
Root N, P, and K contents in different photosynthetic pathway plants in Xinjiang deserts across summer and autumn Note: Figures **(a-c)** show N, P, and K contents in roots of C_3_ and C_4_ plants; **(d-f)** in roots of gymnosperms and angiosperms; and **(g-i)** in roots of different families. m and se represent summer and autumn, respectively. Where C_3_ and C_4_ represent different photosynthetic pathway. CH, Chenopodiaceae; EP, Ephedraceae; LE, Leguminosae; PO, Polygonaceae; SO, Solanaceae; TA, Tamaricaceae; ZY, Zygophyllaceae. Different lowercase letters for different groups in the same column indicate a significant difference in element content (Turkey's HSD test, ANOVA: *p*<0.05).

**Figure 4:**
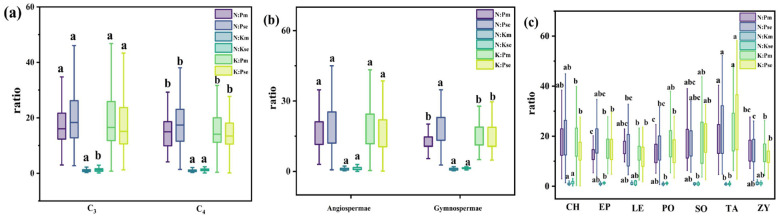
Root N, P, and K ratio of shrubs in different families in Xinjiang desert in summer and autumn Note: Figure **(a-c)** show the ratio of nutrient content in different light and pathway plants, gymnosperms and angiosperms, and plants in different families. Different lowercase letters for different groups in the same column indicate a significant difference in element content (Turkey's HSD test, ANOVA: *p*<0.05).

The concentrations and stoichiometric ratios of N, P, and K were analyzed in the roots of the entire shrub population during summer and autumn using classical statistical methods (**Figures 3**, **4**). The N, P, and K content in the roots of shrub plants in summer were 11.20 ± 0.20 mg g^−^¹, 0.81 ± 0.02 mg g^−^¹, and 12.26 ± 0.24 mg g^−^¹, respectively. In autumn, the root content was 7.91 ± 0.17 mg g^−^¹ for N, 0.49 ± 0.01 mg g^−^¹ for P, and 6.93 ± 0.16 mg g^−^¹ for K. The ratios of N:P, N:K, and K:P for summer roots were 16.47 ± 0.37, 1.21 ± 0.08, and 13.61 ± 0.98, respectively, while the ratios for autumn roots were 20.04 ± 0.64, 2.04 ± 0.32, and 18.64 ± 0.89.

For different functional groups of plants, the mean N, P, and K content in the summer roots of C_3_ plants were 11.47 ± 0.24 mg g^−^¹, 0.81 ± 0.03 mg g^−^¹, and 12.44 ± 0.30 mg g^−^¹, respectively. In contrast, the mean N, P, and K content in the summer roots of C_4_ plants were 10.66 ± 0.35 mg g^−^¹, 0.79 ± 0.03 mg g^−^¹, and 11.89 ± 0.42 mg g^−^¹, respectively. The N and K content in the summer roots of C_3_ plants were significantly higher than those in C_4_ plants. In autumn, the mean N, P, and K content in the roots of C_3_ plants were 8.02 ± 0.22 mg g^−^¹, 0.49 ± 0.02 mg g^−^¹, and 7.11 ± 0.20 mg g^−^¹, respectively. For C_4_ plants in autumn, the mean content values were 7.70 ± 0.26 mg g^−^¹ for N, 0.49 ± 0.07 mg g^−^¹ for P, and 6.54 ± 0.25 mg g^−^¹ for K. There were no significant differences in N, P, and K content between the autumn roots of C_3_ and C_4_ plants. Additionally, the ratios of N:P and K:P in the summer roots of C_3_ plants were significantly higher than those of C_4_ plants. Conversely, the N:P ratio in autumn was significantly lower in C_3_ plants compared to C_4_ plants.

The mean N, P, and K content in the summer roots of angiosperms were 11.10 ± 0.22 mg g^−^¹, 0.78 ± 0.02 mg g^−^¹, and 11.96 ± 0.25 mg g^−^¹, respectively. For gymnosperms, the mean values were 11.93 ± 0.39 mg g^−^¹ for N, 1.00 ± 0.06 mg g^−^¹ for P, and 14.41 ± 0.89 mg g^−^¹ for K. The P and K content in the summer roots of angiosperms were significantly lower than those in gymnosperms. In autumn, the mean N, P, and K content in the roots of angiosperms were 7.81 ± 0.19 mg g^−^¹, 0.49 ± 0.05 mg g^−^¹, and 6.89 ± 0.17 mg g^−^¹, respectively, while the mean values for gymnosperms were 8.58 ± 0.30 mg g^−^¹ for N, 0.53 ± 0.03 mg g^−^¹ for P, and 7.21 ± 0.43 mg g^−^¹ for K. There were no significant differences in nutrient content between the autumn roots of angiosperms and gymnosperms. The ratios of N:P, N:K, and K:P for the summer roots of angiosperms were 16.47 ± 0.37, 1.21 ± 0.08, and 13.61 ± 0.98, respectively, while in autumn, the ratios were 20.04 ± 0.64, 2.04 ± 0.32, and 18.64 ± 0.89. The N:P ratio in summer and the K:P ratios in both summer and autumn were significantly higher in angiosperms compared to gymnosperms.

For different plant families, the N, P, and K content in the autumn roots of *Solanaceae* plants was significantly higher than that of other families. The N content in the summer and autumn roots, as well as the P content in the autumn roots of *Polygonaceae* and *Tamaricaceae* plants, was significantly lower than that of plants from other families. The N:P and K:P ratios in the summer and autumn roots of *Tamaricaceae* plants were significantly higher compared to other families. In contrast, the N:P ratio in the summer roots of *Ephedraceae* and *Polygonaceae* was significantly lower than that observed in other families.

### 3.2 Nutrient resorption efficiency of the desert shrub roots

#### 3.2.1 Root nutrient resorption characteristics of desert shrub plants

**Figure 5:**
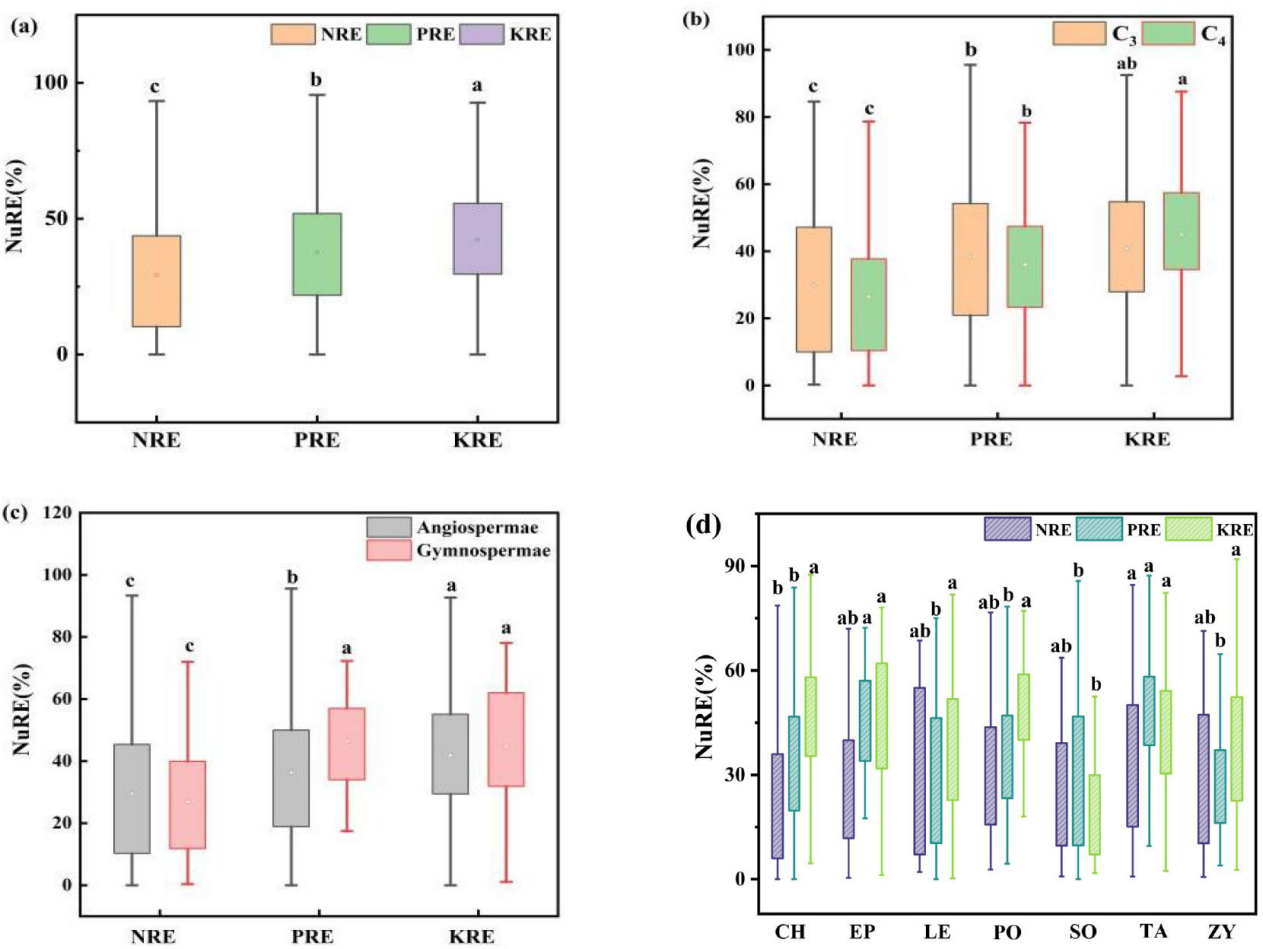
NRE, PRE, and KRE of desert shrub plant roots: **(a)** all plants; **(b)** different light and pathways; **(c)** gymnosperms and angiosperms, **(d)** different families. Where C_3_ and C_4_ represent different photosynthetic pathway. The lowercase letters in the figure indicate significant differences between NRE, PRE, and KRE (Turkey's HSD test, ANOVA: *p*<0.05).

The analysis of N, P, and K nutrient resorption efficiencies in the summer and autumn roots of different functional groups revealed average resorption efficiencies of 29.14 ± 0.98% for NRE, 37.58 ± 0.92% for PRE, and 42.20 ± 0.93% for KRE (**Figure 5**, the resorption characteristics of specific species are shown in **Supplementary Table S3**) (Equation 1). Notably, KRE was significantly higher than PRE and NRE. Among the plant functional groups, C_3_ plants exhibited significantly higher NRE and PRE than C_4_ plants, whereas C_4_ plants had a significantly higher KRE (**Figure 5B**). For angiosperms, the NRE, PRE, and KRE were 29.46 ± 1.06%, 36.31 ± 1.00%, and 41.85 ± 0.98%, respectively, with gymnosperms showing a significantly higher PRE than angiosperms (**Figure 5C**).

The results of N, P and K nutrient resorption efficiencies in the roots of shrubs from different families during summer and autumn are presented in **Figure 5D**, Significant differences in NRE, PRE, and KRE were observed among families (*p*< 0.05). NRE ranged from 24.45% to 33.84%, PRE from 28.20% to 46.41%, and KRE from 22.22% to 47.08%. Notably, *Tamaricaceae* had a significantly higher NRE (33.84%) than *Chenopodiaceae* (24.45%). The PRE of *Ephedraceae* (46.41%) and *Tamaricaceae* (46.23%) were significantly higher than those of other families. In contrast, *Solanaceae* had a significantly lower KRE (22.22%) compared to other families.

#### 3.2.2 Relative resorption efficiency and nutrient limitation of desert shrub roots

The stoichiometric ratio and relative resorption efficiencies are crucial indicators for assessing the nutrient limitations of N and P, and their effectiveness. In summer, the relative resorption efficiency of the roots positively correlated with the ratios N:P_m_, N:K_m_, and K:P_m_ (NRE-PRE (Equation 2) and N:P_m_: r^2^ = 0.03, *p<* 0.5 (**Figure 2A**); NRE-KRE (Equation 3) and N:K_m_: r^2^ = 0.01, *p<* 0.5 (**Figure 2B**); and KRE-PRE (Equation 4) and K:P_m_: r^2^ = 0.03, *p<* 0.5 (**Figure 2C**)). Conversely, in autumn, the relative resorption efficiency in roots demonstrated a consistent negative correlation with the N:P_se_, N:K_se_, and K:P_se_ ratios (NRE-PRE and N:P_se_: r^2^ = 0.31, *p*< 0.01; NRE-KRE and N:K_se_: r^2^ = 0.36, *p<* 0.01 (**Figure 2E**); and KRE-PRE and K:P_se_: r^2^ = 0.25, *p<* 0.01 (**Figure 2F**)). The horizontal black dotted line indicates the position where the NRE equals PRE, and the vertical blue dotted line indicates the corresponding critical N:P ratio. The critical N:P, N:K, and K:P ratios for the summer roots were 38.58, 11.36, and 8.43, respectively, while for the autumn roots were 12.01, 0.60, and 18.85, respectively.

### 3.3 Effects of environmental factors on nutrient resorption efficiency of shrub roots

LMM results (**Table 1**) indicated that both soil total phosphorus (STP) and soil total potassium (STK) had a significant impact on the response variable NRE. Specifically, STP was negatively correlated with NRE, whereas STK was positively correlated. Additionally, pH significantly affected NRE. For PRE, Electrical conductivity (EC), and aridity index (AI) demonstrated highly significant effects, with EC showing a positive correlation and AI a negative correlation. Soil water stress coefficient (K_soil_) also influenced the PRE. For KRE, only STK had a significant effect.

**Table 1:** Responses of all plants' NRE, PRE, and KRE to climate and soil variables.

	Estimate	NRE Std	*p*	Estimate	PRE Std	*p*	Estimate	KRE Std	*p*
pH	-2.86	2.82	0.03^*^	3.13	2.87	0.28	3.86	2.92	0.89
EC	0.09	0.08	0.31	0.26	0.08	0.0009^***^	3.51	7.93	0.66
K_soil_	0.98	1.66	0.23	4.04	1.69	0.017^*^	3.06	1.73	0.08
STN	-0.90	0.88	0.56	-0.56	0.90	0.53	-2.80	9.07	0.76
STP	-3.20	0.84	0.0002^***^	-0.76	0.85	0.37	1.25	8.32	0.14
STK	0.82	0.21	0.0002^***^	0.05	0.22	0.83	4.90	2.22	0.03^*^
MAP	-0.07	0.04	0.09	0.06	0.04	0.20	-6.08	4.05	0.89
MAT	-1.76	1.01	0.08	0.26	1.03	0.80	-1.64	1.03	0.11
AI	-109.40	169.79	0.52	-458.93	172.85	0.008^**^	-2.94	1.77	0.09

Note: Estimate and *p* represent the model regression coefficient and significance level for each explanatory variable, respectively. K_soil_ (%): soil water stress coefficient, defined as the ratio of actual evapotranspiration to potential evapotranspiration; AI (no unit): arid index, calculated as the ratio of precipitation to potential evapotranspiration; drought zone (AI > 0.2), transition zone (0.2< AI< 0.65) and wet zone (AI> 0.65).*p< 0.05;**p< 0.01;***p< 0.001.

For plants in different functional groups (**Table 2**), K_soil_ and AI significantly influenced NRE of C_4_ plants. K_soil_ was negatively correlated with NRE, whereas AI was positively correlated. Additionally, K_soil_ positively affected the NRE of angiosperms. Both K_soil_ and AI significantly affected the PRE of C_3_ plants, with K_soil_ negatively correlated and AI positively correlated. Furthermore, MAT and MAP had significant effects on KRE of C_4_ plants, with negative and positive correlations, respectively.

**Table 2:** Responses of climate and soil variables to NRE, PRE and KRE in different functional groups.

Estimate										
		pH	EC	K_soil_	STN	STP	STK	MAP	MAT	AI
NRE	C_3_	-5.85	0.16	3.81^*^	-2.03	-2.68^**^	0.54^*^	-0.02	-2.46^*^	-449.14^*^
	C_4_	13.71^*^	-0.48^*^	-16.71^***^	4.15^*^	-2.57	0.75	-0.07	-2.83	1689.60^***^
	An	-3.33	0.05	1.90	-2.04^*^	-2.38^**^	0.81^***^	-0.01	-1.64	-261.27
	Gy	-12.72	0.58	-18.46	4.73	-0.69	-0.78	-0.22	-3.46	2060.58
PRE	C_3_	1.60	0.16	5.43^**^	0.76	-1.05	0.26	0.10^*^	0.17	-689.01^***^
	C_4_	4.68	0.39	0.39	-2.02	0.52	-0.51	-0.07	-0.81	740.90
	An	2.47	2.48^**^	3.47^*^	-2.32^*^	3.48	1.27	9.48	1.17	-4.14^*^
	Gy	-2.01	2.89	-3.10	6.38	2.39	-1.58	-1.41	-1.70	2.91
KRE	C_3_	-7.68^*^	5.72	3.69^*^	9.68	1.15	6.14^*^	1.24^*^	-2.97^*^	-4.72^*^
	C_4_	1.42^*^	2.15	-2.69	-4.03^*^	6.61	2.28	-7.75^***^	9.01^***^	6.38
	An	2.32	5.88	2.30	-1.41	1.80^*^	4.30	-1.23	-1.14	-2.00
	Gy	-1.84	-2.43	5.07	7.90	-8.61	6.78	1.14	-9.32	-4.69

C_3_ and C_4_ represent plants with different light and pathway, respectively; An and Gy represent Angiospermae and Gymnospermae, respectively. *p< 0.05; **p< 0.01; ***p< 0.001.

Among different families (**Table 3**) NRE of *Chenopodiaceae* and *Polygonaceae f*amilies exhibited significant responses to various environmental factors. Among these, K_soil_, Soil total nitrogen (STN), and MAT had the most pronounced effects on the NRE of *Chenopodiaceae* plants. In contrast, pH, K_soil_, STN, STK, MAP, and MAT significantly affected the NRE of *Polygonaceae* plants. In addition, K_soil_, STK, MAT, and AI had the most significant effects on the PRE of *Polygonaceae* plants. MAP and AI significantly affected the KRE of *Polygonaceae* plants.

**Table 3:** Responses of climate and soil variables to NRE, PRE and KRE of different families.

	Family	Estimate								
		pH	EC	K_soil_	STN	STP	STK	MAP	MAT	AI
NRE	CH	7.05	-2.65	1.83^***^	-6.19^***^	5.78	1.14^***^	-1.04	8.47^***^	1.59^**^
	EP	-2.31^*^	6.69^*^	-3.48^*^	8.87^*^	-3.66	-1.11	-1.31^*^	-1.35^*^	3.38^**^
	LE	2.93	7.12	-1.15^**^	4.80	-3.08	3.21	-1.11^**^	-3.20^**^	1.03^**^
	PO	-5.15^***^	-1.73	-3.51^***^	8.93^***^	-7.27^**^	-2.92^***^	4.43^***^	-2.86^***^	2.47^**^
	SO	-4.53	-1.51	1.53	-7.38	3.75^***^	-5.82	5.54	5.75	-4.36
	TA	-8.89	3.89	-8.68	-7.55	3.16	-1.27	6.12^***^	-2.44^***^	-4.71
	ZY	6.36^*^	-2.03	-1.15^***^	2.91	-2.33	1.84	-1.13	2.29	1.23^***^
PRE	CH	4.54	1.77	1.41^*^	-3.47^*^	-4.52	-6.36	7.25	2.34	-1.26^*^
	EP	-1.13	3.60	-4.17^**^	7.53^*^	3.19	-1.39	8.17	-1.81^*^	3.75^**^
	LE	2.40	3.09	-6.77	5.92	-2.06	2.14	9.15	-2.20	5.25
	PO	-3.14^*^	5.44	-4.41^***^	7.19^**^	-3.52	-3.05^***^	-2.50	-1.78^***^	4.18^***^
	SO	-5.62	-6.08^**^	3.87	3.61	1.58	-9.27^**^	6.00	4.85	-1.11^*^
	TA	2.21^*^	5.24	-1.63^**^	5.99	-1.51	1.91^*^	-5.74	5.17	1.73^**^
	ZY	-2.40	-3.66	4.48	-2.06	1.90	-1.43	1.20	-1.23	-1.03
KRE	CH	3.06	2.99	1.42^*^	-2.89	2.37	7.59	-1.30	1.51	-1.30^*^
	EP	-1.92	-4.84	-1.14	9.38^*^	-9.17	-2.60	2.32	-1.19	6.32
	LE	-3.51	1.41	-8.33	-3.21	-3.44	-5.04	9.26	1.09	6.05
	PO	-2.13	2.71	-2.22^*^	2.20	-1.22	-2.60	-6.53^**^	2.64	2.75^**^
	SO	-4.54	-2.51	2.75	-7.67	2.23^*^	-8.90^*^	9.58^*^	8.55^*^	-6.61
	TA	1.44	-2.45	-1.48^*^	1.14	3.89	4.57	3.86^*^	8.51	1.36
	ZY	-2.15	3.92^*^	-1.78	-7.89	8.44	-1.74	-1.47	-9.03	1.45

CH, Chenopodiaceae; EP, Ephedraceae; LE, Leguminosae; PO, Polygonaceae; SO, Solanaceae; TA, Tamaricaceae; ZY, Zygophyllaceae. *p< 0.05; **p< 0.01; ***p< 0.001.

**Figure 6:**
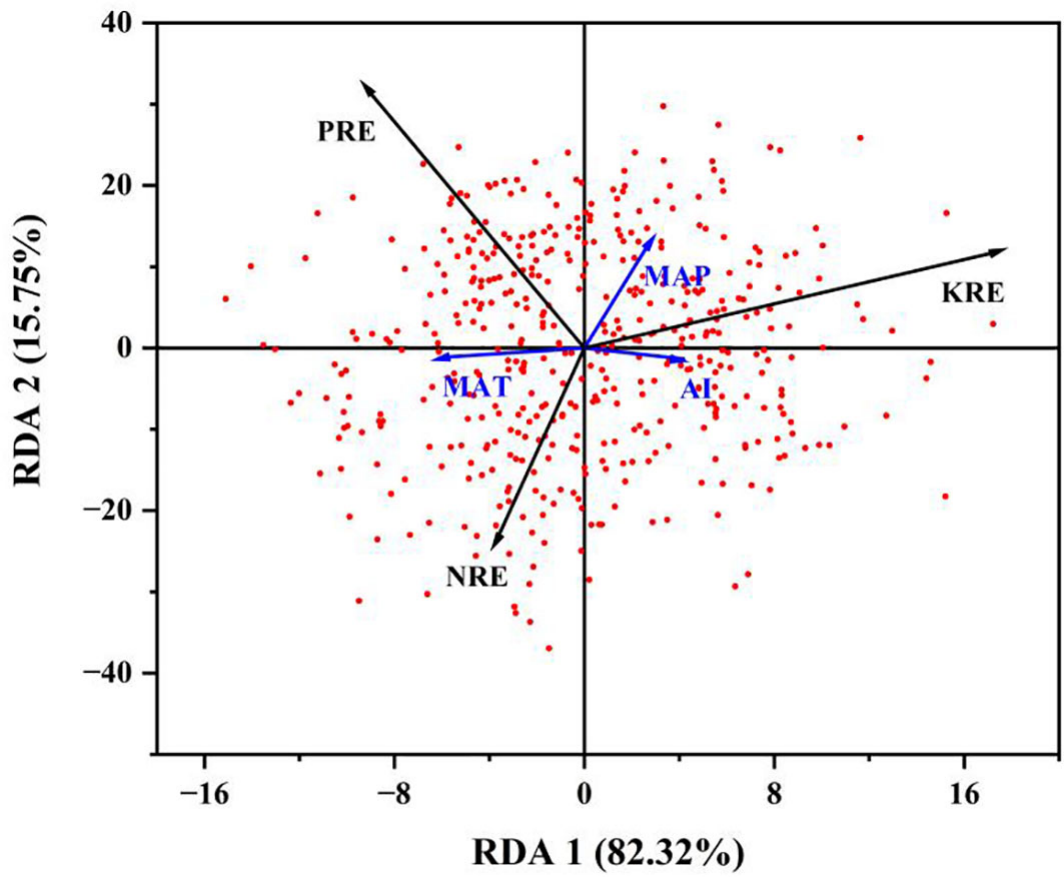
The influence of climatic factors on nutrient resorption efficiency (RDA analyse). Note: The direction of the blue arrows in the figure indicates the changing trend of the environmental factor, and the length of the arrows indicates the degree to which this environmental factor explains the sample distribution. The longer the arrow indicates that the greater the influence of this environmental factor on the sample distribution. The angles between the arrows and the sorting axes indicate the correlation between the environmental factors and the sorting axes. The smaller the clip angle, the higher the correlation. The quadrant in which the arrow indicates the positive or negative correlation of environmental factors with the sorting axis. The points in the plot represent species at different sampling points. The locations of the dots shows their relative positions in the RDA analysis.

The relationship between climate and nutrient resorption efficiency was illustrated in **Figure 6**. The RDA results showed that the first axis (RDA 1) explained 82.32% of the variation, while the second axis (RDA 2) explained 15.75% of the variation. Overall, the first two axes accounted for over 98% of the ecological data variation, indicating that the selected environmental variables significantly explained species distribution. RDA 1 was primarily driven by MAP and MAT, suggesting that under warm and humid conditions, plants exhibited significantly higher PRE and KRE. RDA 2 was closely related to the AI, implying that high soil nitrogen availability reduced NRE.

## 4 Discussion

### 4.1 Stoichiometric characteristics and stoichiometric ratios of N, P, and K in desert shrub roots

N and P are essential components of important biomolecules such as proteins, nucleic acids, and amino acids in plants. K plays important physiological regulatory functions in plant cells (Lopez et al., 2023; Das and Mondal, 2024). The concentrations of these elements not only reflect the inherent physiological characteristics of plants but also illustrate their adaptive capacity and response mechanisms to environmental conditions (Han et al., 2011; An et al., 2021). In the present study, the average concentrations of N, P, and K in the roots of all shrub species during summer were significantly higher than in the roots during autumn. This difference is primarily attributed to the active growth and metabolism of roots during summer, in the summer high temperature and drought conditions, plants through a series of adaptive mechanisms to maintain growth, these mechanisms include the adaptability of root changes, antioxidant defense and metabolic regulation, hormone signal regulation and microbial interaction (Gupta et al., 2021; Zhang et al., 2022), leading to an increased demand for nutrients and enhanced ability to absorb nutrients from the soil (Moreau et al., 2019). With root senescence, metabolic activity declines, nutrient demand decreases, and elemental concentrations diminish. The concentration of N and P in the roots of desert plants in summer was higher than both global (8.8 mg g^−1^, 0.69 mg g^−1^) and national levels (4.87 mg g^−1^, 0.47 mg g^−1^) (Yuan et al., 2011; Yang et al., 2018). Desert plants sustain their normal physiological functions by absorbing sufficient water and nutrients, allowing them to adapt to arid and nutrient-poor environments (Schenk and Jackson, 2002; Kautz et al., 2013; Mahdieh et al., 2021; Tariq et al., 2024). In summer, the K concentration in roots was lower than that in other regions, which may be attributed to the specific characteristics of desert habitats. Drought and saline conditions limit K uptake by plants. C_3_ plants rely heavily on ribulose-1,5-bisphosphate carboxylase/oxygenase (Rubisco) for photosynthesis, an enzyme that is nitrogen-rich. This reliance means C_3_ plants need higher nitrogen levels to support Rubisco synthesis and function. However, Rubisco’s susceptibility to oxygen inhibition during CO_2_ fixation leads to photorespiration, which reduces photosynthetic efficiency. To compensate, C_3_ plants require more nitrogen (Paulus et al., 2013; Li et al., 2024a; Wang F. et al., 2020). Additionally, C_3_ plants often have a higher specific root length, allowing them to explore soil more extensively for nutrients like N and K, which are essential for photosynthesis and maintaining cell osmotic pressure (Mukundan et al., 2024). In contrast, C_4_ plants use the more efficient enzyme phosphoenolpyruvate carboxylase (PEP carboxylase) for initial CO_2_ fixation, reducing their dependence on Rubisco and lowering their nitrogen requirements. Consequently, C_4_ plants have relatively lower nitrogen concentrations in their roots compared to C_3_ plants (Huang et al., 2022), C_4_ plants are generally more adaptable to high temperatures and drought conditions, which may contribute to the observed differences in physiological and ecological performance between C_4_ and C_3_ plants (Ozeki et al., 2022). C_4_ plants often exhibit greater productivity and resilience in extreme environments, thereby giving them a competitive advantage over C_3_ plants in certain habitats (Guidi et al., 2019). Gymnosperms preferentially utilize available N, P, and K during biosynthesis, leading to higher concentrations of these essential nutrients in their tissues. This selective uptake can be attributed to the phosphoenolpyruvate (PEP) synthesis pathway and adaptive changes in the cell wall, Under drought conditions, lignin and phenolic compounds content in gymnosperm cell walls increase, which contribute to cell wall hardening and lignification, thus improving plant drought resistance, which optimize nutrient assimilation and utilization (Wang et al., 2019; Langguth et al., 2024). In resource-limited environments, *Only Tamariaceae* shows the preferential absorption of K^+^ to support osmotic regulation and water retention, and this adaptability is mainly attributed to its efficient K^+^ absorption mechanism. *Tamariaceae* plants absorb and transport K^+^ through high-affinity K^+^ transporters (e. g., HAK 5) and K^+^ channels (such as AKT 1). These transporters exhibit efficient absorption under low K conditions, thus helping plants maintain cellular osmotic pressure and water balance in drought and salt stress environments (Chen et al., 2023). Conversely, *Chenopodiaceae* plants can adjust the osmotic levels in their cells by regulating the concentrations of N and P, thereby enhancing their adaptability to extreme environment (He et al., 2024).

The nitrogen to phosphorus (N:P) ratio in terrestrial vegetation serves as an indicator of soil nitrogen and phosphorus availability, highlighting nutrient limitations and the overall health of plant growth (Güsewell, 2004). Compared to leaves, the N:P ratio of stems and roots can serve as a more effective indicator of soil nutrient availability (Schreeg et al., 2014; Zeng et al., 2016). This study found that the N:P ratio of mature roots of all shrubs was 16.47 ± 0.37, while that of senescent roots was 20.04 ± 0.64. Both ratios exceeded 16, suggesting that the growth of desert shrub roots in Xinjiang may be limited by P. With limited water and nutrients, desert plants have reduced resource absorption, leading to decreased nutrient cycling and metabolic activity (Li et al., 2024a). The K:P ratio of the desert shrub roots was 18.41, further indicating that the growth of desert shrubs in Xinjiang was limited by P, which is consistent with the results of our previous study in this region (Luo et al., 2020). Because of their complex metabolic pathways, angiosperms can absorb and utilize N and K from the soil more effectively, resulting in higher N:P and K:P ratios. For example, they can dissolve the P in the soil through root exudates (such as organic acids and phosphatases), thus improving phosphorus availability (Ma et al., 2022; Pang et al., 2024). The root structure of angiosperms and microbial symbiosis (such as mycorrhizal fungi) also enhance N and K resorption. In addition, Angiosperms are more efficient in absorbing N and K by optimizing root architecture and increasing nutrient uptake efficiency (Langguth et al., 2024). *Tamarixaceae* plants exhibit high K:P ratios and possess well-developed roots that can penetrate deep into the soil to access water and nutrients, particularly in arid saline environments where their capacity for K uptake is enhanced (Hussain et al., 2022; Islam et al., 2024).

### 4.2 Changes in nutrient resorption characteristics of desert shrub roots

#### 4.2.1 Characteristics of NuRE

In desert ecosystems, the harsh environmental conditions typically lead to lower rates of nutrient resorption and utilization compared to other ecosystems. As a result, desert plants are more reliant on internal nutrient cycling for their survival (Reichert et al., 2022; Tariq et al., 2024). In the present study, we found that the nutrient resorption efficiencies of the desert shrub roots were 29.14% for nitrogen (NRE), 37.58% for phosphorus (PRE), and 42.20% for potassium (KRE), as shown in **Figure 1**. These values are higher than the global average NRE of 27% for roots but lower than the global average PRE of 57% (Freschet et al., 2010). This discrepancy may be attributed to the fact that desert plants thrive in arid, nutrient-poor soils, and are developmentally and physiologically adapted to low-nutrient conditions (Suseela and Tharayil, 2018). In a study of root P levels, Yuan et al. (2011) reported that senescing roots reabsorbed 27% of P on average. Additionally, in a study involving 40 subarctic species, Freschet et al. (2010) found that 57% of P was resorbed in the roots, which was comparable to the 63% resorption rate observed in the leaves. The higher KRE value may reflect the need for plants in desert ecosystems to adapt to drought and water scarcity. To cope with these conditions, plants enhance their K resorption mechanisms, thereby improving K use efficiency.

The NRE and PRE in C_3_ plants were generally higher than those of C_4_ plants, a phenomenon that is attributable to the relatively low photosynthetic efficiency of C_3_. This inherent inefficiency coerces C_3_ plants to invest greater amounts of N and P to sustain their photosynthetic processes and metabolic functions (Kubásek et al., 2013; Guidi et al., 2019; Tanigawa et al., 2024). The structural simplicity of the xylem vascular bundle in gymnosperms enhances their ability to efficiently resorb and transport essential nutrients, optimizing nutrient resorption and retention, thereby facilitating their adaptability to various environmental conditions (Gleason et al., 2016; Langguth et al., 2024). *Tamaricaceae* plants exhibited a stronger absorption capacity for N and P, probably because their roots form symbiotic relationships with N-fixing bacteria (Cavagnaro, 2016). These bacteria convert N into a form that can be readily absorbed by plants, thereby enhancing the N utilization efficiency. In addition, organic acids secreted by *Tamaricaceae* plants, such as citric and oxalic acids, converts P in the soil into a form that can be absorbed by plants (Kumar and Rai, 2020), thereby increasing the effective uptake and utilization of P. In contrast, the K absorption efficiency of *Solanaceae* plants was significantly lower than that of other families. This may be attributed to the higher degree of lignification in their roots and increased rigidity of their cell walls, which adversely affects the extension and branching capabilities of the roots, consequently affecting their efficiency in absorbing water and nutrients (Basak et al., 2024).

#### 4.2.2 Relative resorption efficiency and nutrient limitation

Nutrient ratios and relative nutrient resorption are important indicators for assessing plant nutritional status and understanding potential mechanisms of nutrient resorption (Güsewell, 2005; Schreeg et al., 2014). In the present study, the root PRE of desert shrub plants was higher than the NRE. Specifically, the relative resorption efficiencies of N and P (NRE-PRE< 0) were negative, which aligns with the prediction of the relative resorption hypothesis and suggests that plants tend to absorb limiting nutrients more than other nutrients (Han et al., 2013). The NRE-PRE, NRE-KRE, and KRE-PRE of all shrub roots were positively correlated with the N:P, N:K, and K:P ratios in roots during summer, whereas they were negatively correlated with these ratios in autumn (**Figure 3**), which is also consistent with our previous research results. When the N:P, N:K, and K:P ratios were 12.01, 0.60, and 18.85, respectively, the absorption efficiencies of all shrub roots in autumn reached equilibrium (NRE = PRE, NRE = KRE, and KRE = PRE), indicating that these plant groups were limited by N and P, N and K, and K and P near these critical ratios.

### 4.3 Relationship between nutrient resorption characteristics and environmental factors

Changes in the nutrient resorption efficiency reflect the response and adaptation of plants to their environment (Hartley et al., 2007; Liu et al., 2010; Takahashi et al., 2020). Desert plants have developed specialized survival and nutrient utilization strategies, through long-term adaptation, to cope with drought and high-salinity conditions (Gao et al., 2023). LMM results showed an inverse relationship between NRE and increased STP and STK in all plants. Soil N concentration in desert areas, characterized by low precipitation and high evaporation rates, is typically low. This limitation inhibits vegetation growth and intensifies rock weathering, increasing P concentration (Delgado-Baquerizo et al., 2013). Soil erosion further reduces plant coverage, which in turn inhibits the N mineralization process in the soil, decreasing the available N concentration. Excessive P occupies a significant number of absorption sites in plant roots, thereby affecting N uptake (McCulley et al., 2004). Soil K exerts a strong influence on N uptake by plant roots. As STK levels increase, the activity and absorption capacity of plant roots also increases, potentially enhancing NRE. The NRE of C_4_ plants decreased with increasing K_soil_ and increased with increasing AI. This phenomenon may be attributed to the inhibition of photosynthesis and growth in C_4_ plants as drought conditions intensify and soil moisture decreases, leading to reduced absorption capacity (Guidi et al., 2019). The NRE of *Chenopodiaceae* and *Polygonaceae* families exhibited the most significant responses to environmental factors. These two families dominate desert alkaline and arid regions and possess strong salt and drought tolerances (Gross et al., 2024). Plants accumulate compatible solutes, such as proline and betaine, in cells under high-salt and drought conditions. Proline is an important osmotic regulator that increases the osmotic pressure of cells, enabling them to absorb water from the outside environment under low-water-potential conditions (Li et al., 2024b). Their specialized survival and nutrient utilization strategies enable them to respond rapidly to environmental changes and sustain their growth under water-scarce conditions.

PRE in all plants roots increased with increasing EC but decreased with increasing drought severity. An increase in EC elevates the ion concentration in the soil solution, allowing plants to access various nutrients necessary for adaptation to their growth environment more readily (Zhao et al., 2015). Conversely, plants experience water deficiency stress as drought conditions worsens. The limited mobility of nutrients in arid soils affects the concentration of nutrients absorbed by plants (Gao et al., 2022). The PRE of C_3_ plants decreased with increasing K_soil_ and increased with increasing AI. Under water stress, C_3_ plants close their stomata to reduce water evaporation, which negatively affects photosynthesis and metabolic activities of cells (Mukundan et al., 2024). Consequently, the entire plant reduces the demand and utilization of P. Under water stress conditions, P exhibits poor mobility in the soil, primarily exists in the form of insoluble compounds (Salim et al., 2024), its uptake by plant roots is restricted, and resorption efficiency reduces. Unique physiological structure and characteristics of *Polygonaceae* plants make them highly sensitive to variations in soil and climatic conditions. Their root systems are well-developed in arid and saline–alkaline environments, with extensive root hairs and a complex branching structure, which can effectively explore a larger soil volume to absorb water and nutrients (Vries et al., 2019). All these factors, including the root system, stomatal regulation, photosynthetic efficiency, and physiological adaptability, together enhance the nutrient and water resorption efficiency of *Polygonaceae* plants, thereby improving their survival and ecological competitiveness in harsh environments (McCulley et al., 2004; Freschet and Roumet, 2017; Moreau et al., 2019).

STK plays a crucial role in predicting the changes in the potassium resorption efficiency (KRE) of entire plants, encompassing both the soluble forms that plants can directly absorb and the insoluble forms released from soil minerals. In desert soils, the high potassium availability promotes plant growth and enhances interactions with microorganisms, leading to improved potassium utilization efficiency by plants (Cavagnaro, 2016). MAP and AI significantly influenced the KRE in *Polygonaceae* plants. Precipitation and drought conditions affect plant growth and nutrient uptake by affecting soil moisture and nutrient availability (Luo et al., 2024). Excessive precipitation can result in K loss, whereas moderate drought conditions can enhance K absorption by plants (Lebaudy et al., 2007). The key factors that have a significant impact on KRE in *Polygonaceae* plants are adaptability and regulatory mechanisms under diverse water conditions (Yang et al., 2020). Under arid or water-limited conditions, these plants have evolved specific morphological and physiological traits to cope with water scarcity. For instance, they may develop more efficient water-absorbing root systems or adjust their stomatal conductance to minimize water loss while maintaining essential physiological processes related to K uptake and utilization (Adame et al., 2021). Root branching and extension are key features of plant adaptation to drought stress (Zhang et al., 2021). In drought environments with limited resources, the branching structure of the roots can significantly increase the contact area between the roots and the soil, thereby allowing for more effective exploration of soil nutrients (Gao et al., 2024). Studies have shown that moderate drought stress can promote the formation of root branches and the lateral extension of roots (Shafi et al., 2024). This change in root architecture enables plants to more efficiently absorb potassium ions from the soil under drought conditions, especially when potassium availability in the soil is low.

Drought stress significantly constrains the physiological and ecological processes of desert plants. Redundancy analysis (RDA) revealed that MAP, MAT, and AI collectively explained over 98% of the variation in nutrient resorption efficiency, underscoring the pivotal role of water availability in regulating plant nutrient cycling under extreme environmental conditions. This study demonstrates distinct response mechanisms among different nutrient elements in terms of resorption efficiency. Phosphorus resorption efficiency (PRE) exhibited a precipitation-driven response signature. Episodic rainfall events in arid environments temporarily alleviated soil water deficits, significantly enhancing phosphorus uptake during the growth period (Zhao M. et al., 2024). Elevated MAT prolonged the root nutrient resorption and enzymatic reaction cycles, enabling plants to accumulate phosphorus reserves in mature leaves, thereby forming a vital allocation mechanism for scarce phosphorus resources. This adaptive strategy manifested as a marked increase in PRE under warm and humid conditions (Cao et al., 2024). Potassium resorption efficiency (KRE) reflected drought adaptation strategies. The enhancement of KRE was directly linked to osmotic regulation demands, as plants under drought stress intensified potassium resorption to maintain cell membrane integrity (Lee et al., 2024). The negative correlation between AI and NRE highlighted the inhibitory effects of prolonged drought on nitrogen conservation. High AI values exacerbated soil water deficits, suppressing microbial-mediated nitrogen mineralization processes and forcing plants to prioritize metabolic resource allocation (Shafi et al., 2024; Li et al., 2024b). Our results show that the efficiency of nutrient resorption is influenced by both soil and climatic factors. Temperature affects nutrient resorption by impacting soil nutrient availability, the rate of plant senescence, and nutrient resorption by altering soil moisture conditions and nutrients levels (Yuan et al., 2011; Cao et al., 2020). The nutrient cycling process of plants in desert ecosystems is complex, and the primary factors that influence nutrient resorption warrant further investigation.

## 5 Conclusion

This study comprehensively analyzes the variations in nutrient stoichiometry and resorption patterns of desert shrub roots in Xinjiang and their responses to climate and soil factors. The results indicate that nitrogen (N) and phosphorus (P) concentrations in desert shrub roots are significantly higher than those in other regions, while potassium (K) concentrations are significantly lower. The N:P ratio exceeds 16, suggesting that growth may be limited by P availability. Additionally, KRE in desert shrub roots significantly surpasses both NRE and PRE, highlighting the importance of K in their nutrient resorption strategies. Soil moisture, nutrient content and its chemical form will affect the nutrient absorption and utilization of desert plants, and climatic factors such as temperature and precipitation will affect soil moisture, nutrient availability and plant metabolic process, thereby affecting the efficiency of plant nutrient resorption. The findings provide important insights into the resorption patterns and utilization strategies of root nutrients in desert shrubs, as well as the relationship between plants and their environment within desert ecosystems.

## Data Availability

The raw data supporting the conclusions of this article will be made available by the authors, without undue reservation.
