# Supplementary material for: Soil and climate factors affect the nutrient resorption characteristics of desert shrub roots in Xinjiang, China
Source: Front Plant Sci. 2025 Jun 27;16:1518846. doi: 10.3389/fpls.2025.1518846 (PMC12247178; doi:10.3389/fpls.2025.1518846)
Supplement: Supplementary file 1 [file Table1.docx]

**Supporting information**

**Table S1** Location information and climatic characteristics of sampling sites in desert areas in Xinjiang, China

**Table S2** Information of the species sampled in the desert shrub of Xinjiang, China.

**Table S3** Resorption characteristics of N, P and K nutrients in different species of desert shrubs in Xinjiang

**Table S4** Soil characteristics in the desert sites in Xinjiang, China.

**Table S1** Location information and climatic characteristics of sampling sites in desert areas in Xinjiang, China

| Site | LON | LAT | ALT | MAT | MAP | AI |
| --- | --- | --- | --- | --- | --- | --- |
| 1 | 80.66 | 40.75 | 1024 | 11.41 | 53 | 0.04 |
| 2 | 80.82 | 41.25 | 984 | 10.72 | 83 | 0.07 |
| 3 | 81.05 | 40.24 | 1025 | 11.87 | 45 | 0.03 |
| 4 | 81.09 | 41.4 | 1024 | 10.95 | 94 | 0.08 |
| 5 | 81.24 | 40.43 | 975 | 11.5 | 50 | 0.03 |
| 6 | 81.77 | 40.61 | 984 | 11.43 | 41 | 0.03 |
| 7 | 81.94 | 40.68 | 980 | 11.58 | 70 | 0.03 |
| 8 | 82.11 | 40.82 | 973 | 11.62 | 45 | 0.04 |
| 9 | 82.44 | 41.61 | 997 | 11.09 | 138 | 0.06 |
| 10 | 82.59 | 41.67 | 1031 | 11.18 | 135 | 0.06 |
| 11 | 82.78 | 41.91 | 1110 | 9.54 | 167 | 0.09 |
| 12 | 83.02 | 41.95 | 1451 | 9.12 | 169 | 0.09 |
| 13 | 83.3 | 41.52 | 958 | 11.6 | 118 | 0.04 |
| 14 | 83.49 | 44.57 | 365 | 9.4 | 159 | 0.11 |
| 15 | 83.56 | 46.43 | 480 | 6.96 | 226 | 0.23 |
| 16 | 84.2 | 41.34 | 896 | 11.38 | 47 | 0.04 |
| 17 | 84.3 | 41.58 | 912 | 11.04 | 54 | 0.05 |
| 18 | 84.72 | 45.18 | 270 | 8.7 | 118 | 0.11 |
| 19 | 84.86 | 44.85 | 295 | 9.63 | 127 | 0.11 |
| 20 | 85.03 | 45.29 | 270 | 8.89 | 114 | 0.1 |
| 21 | 85.15 | 45.72 | 366 | 9.07 | 122 | 0.11 |
| 22 | 85.23 | 41.36 | 916 | 11.5 | 64 | 0.04 |
| 23 | 85.39 | 45.92 | 307 | 9.2 | 128 | 0.11 |
| 24 | 85.86 | 46.24 | 526 | 7.94 | 159 | 0.13 |
| 25 | 86.16 | 41.18 | 878 | 11.73 | 52 | 0.04 |
| 26 | 86.27 | 44.48 | 365 | 7.26 | 141 | 0.13 |
| 27 | 86.59 | 44.26 | 470 | 9.36 | 152 | 0.14 |
| 28 | 86.89 | 47.64 | 522 | 5.29 | 154 | 0.17 |
| 29 | 88.19 | 42.26 | 892 | 10.04 | 75 | 0.07 |
| 30 | 88.3 | 44.52 | 499 | 7.63 | 169 | 0.16 |
| 31 | 88.34 | 43.4 | 1196 | 5.95 | 173 | 0.19 |
| 32 | 88.38 | 44.95 | 665 | 6.38 | 180 | 0.18 |
| 33 | 88.64 | 42.87 | 290 | 14.35 | 38 | 0.03 |
| 34 | 89 | 44.94 | 571 | 8.05 | 146 | 0.16 |
| 35 | 90.06 | 44.81 | 563 | 8.68 | 109 | 0.12 |
| 36 | 90.14 | 44.18 | 756 | 6.86 | 110 | 0.12 |
| 37 | 90.54 | 44.19 | 870 | 6.61 | 91 | 0.1 |
| 38 | 90.56 | 44.05 | 958 | 5.85 | 94 | 0.1 |
| 39 | 90.82 | 44.41 | 905 | 7.39 | 83 | 0.09 |
| 40 | 91.03 | 44.8 | 758 | 6.79 | 124 | 0.12 |
| 41 | 91.14 | 44.18 | 758 | 6.77 | 120 | 0.12 |
| 42 | 91.33 | 44.76 | 766 | 8.14 | 70 | 0.07 |

Note: LON (°), longitude; LAT (°), latitude; ALT (km), altitude; MAT (℃), mean annual temperature; MAP (mm), mean annual precipitation; AI (unitless), aridity index, defined as the ratio of precipitation to potential evapotranspiration.

**Table S2** Information of the species sampled in the desert shrub of Xinjiang, China.

| Species | Family | | photosynthetic pathway | Phylogeny |  |
| --- | --- | --- | --- | --- | --- |
| *Atraphaxis bracteata* | Polygonaceae | | C_3_ | Angiospermae |  |
| *Atraphaxis virgata*  *Calligonum ebi-nuricum*  *Calligonum junceum*  *Calligonum leucocladum*  *Caragana acanthophylla*  *Caragana aciphylla*  *Caragana dasyphylla*  *Ephedra intermedia*  *Ephedra przewalskii*  *Halimodendron halodendron*  *Halostachys caspica*  *Haloxylon ammodendron*  *Krascheninnikovia ewersmanni* | | | Polygonaceae  Polygonaceae  Polygonaceae  Polygonaceae  Leguminosae  Leguminosae  Leguminosae  Ephedraceae  Ephedraceae  Leguminosae  Leguminosae  Chenopodiaceae  Chenopodiaceae | C_3_  C_4_  C_4_  C_4_  C_3_  C_3_  C_3_  C_3_  C_3_  C_3_  C_3_  C_4_  C_3_ | Angiospermae  Angiospermae  Angiospermae  Angiospermae  Angiospermae  Angiospermae  Angiospermae  Gymnospermae  Gymnospermae  Angiospermae  Angiospermae  Angiospermae  Angiospermae |
| *Lycium ruthenicum* | Solanaceae | | C_3_ | Angiospermae |  |
| *Nitraria sphaerocarpa*  *Nitraria tangutorum* | Zygophyllaceae  Zygophyllaceae | | C_3_  C_3_ | Angiospermae  Angiospermae |  |
| *Tamarix arceuthoides* | Tamaricaceae | | C_3_ | Angiospermae |  |
| *Tamarix hispida* | Tamaricaceae | | C_3_ | Angiospermae |  |
| *Tamarix ramosissima* | Tamaricaceae | | C_3_ | Angiospermae |  |
| *Zygophyllum xanthoxylon* | Zygophyllaceae | | C_4_ | Angiospermae |  |

**Table S3 Resorption characteristics of N, P and K nutrients in different species of desert shrubs in Xinjiang**

| **Species** | **n** | **NRE(%)** | **PRE(%)** | **KRE(%)** |
| --- | --- | --- | --- | --- |
| *Atraphaxis bracteata* | 3 | 41.33 | 34.36 | 42.79 |
| *Atraphaxis virgata* | 3 | 79.93 | 43.67 | 43.86 |
| *Calligonum ebi-nuricum* | 3 | 30.73 | 17.48 | 21.17 |
| *Calligonum junceum* | 10 | 23.62 | 27.81 | 41.13 |
| *Calligonum leucocladum* | 36 | 31.21 | 43.37 | 50.11 |
| *Caragana acanthophylla* | 18 | 29.43 | 14.44 | 45.99 |
| *Caragana aciphylla* | 6 | 57.61 | 36.06 | 36.78 |
| *Caragana dasyphylla* | 5 | 4.37 | 49.67 | 37.00 |
| *Ephedra intermedia* | 25 | 21.88 | 44.51 | 48.92 |
| *Ephedra przewalskii* | 32 | 30.79 | 47.89 | 41.37 |
| *Halimodendron halodendron* | 11 | 42.36 | 32.57 | 55.56 |
| *Halostachys caspica* | 33 | 32.62 | 32.29 | 43.73 |
| *Haloxylon ammodendron* | 74 | 22.17 | 38.15 | 47.10 |
| *Krascheninnikovia ewersmanni* | 10 | 14.38 | 14.89 | 52.36 |
| *Lycium ruthenicum* | 35 | 25.64 | 28.82 | 22.22 |
| *Nitraria sphaerocarpa* | 16 | 33.37 | 32.22 | 37.76 |
| *Nitraria tangutorum* | 11 | 20.61 | 24.66 | 53.68 |
| *Tamarix arceuthoides* | 8 | 49.69 | 43.53 | 58.81 |
| *Tamarix hispida* | 21 | 36.37 | 42.74 | 34.97 |
| *Tamarix ramosissima* | 79 | 31.56 | 47.43 | 42.81 |
| *Zygophyllum xanthoxylon* | 16 | 35.04 | 27.03 | 25.11 |
| total | 455 | 29.14 | 37.58 | 42.20 |

**Table S4** Soil characteristics in the desert sites in Xinjiang, China.

| Site | STN | STP | STK | pH | EC | Ksoil |
| --- | --- | --- | --- | --- | --- | --- |
| 1 | 0.86 | 0.49 | 22.24 | 8.6 | 18.04 | 4.07 |
| 2 | 1.3 | 0.53 | 21.71 | 8.33 | 12.55 | 6.74 |
| 3 | 4.6 | 6.5 | 16.88 | 9.23 | 2.78 | 3.16 |
| 4 | 1.19 | 0.41 | 11.12 | 9.27 | 5.31 | 7.57 |
| 5 | 1.24 | 2.45 | 23.41 | 8.75 | 6.27 | 3.32 |
| 6 | 0.18 | 0.01 | 32.64 | 8.11 | 8.51 | 3.32 |
| 7 | 1.78 | 0.95 | 27.12 | 8.47 | 4.62 | 3.41 |
| 8 | 0.23 | 0.02 | 26.98 | 8.13 | 3.51 | 3.66 |
| 9 | 2.93 | 3.06 | 21.35 | 8.53 | 8.09 | 5.74 |
| 10 | 0.59 | 0.21 | 23.19 | 8.32 | 4.36 | 5.74 |
| 11 | 1.84 | 2.56 | 22.41 | 8.56 | 7.19 | 9.32 |
| 12 | 0.92 | 0.43 | 23.79 | 8.71 | 1.56 | 9.57 |
| 13 | 2.63 | 0.68 | 20.4 | 8.83 | 44.79 | 4.41 |
| 14 | 1.89 | 2.82 | 25.2 | 8.6 | 5.99 | 11.3 |
| 15 | 0.28 | 0.1 | 30.09 | 9.95 | 6.89 | 22.87 |
| 16 | 0.19 | 0.04 | 23.06 | 8.15 | 3.7 | 3.99 |
| 17 | 0.23 | 0.06 | 27.35 | 8.09 | 41.27 | 4.82 |
| 18 | 1.36 | 0.76 | 11.91 | 8.28 | 0.91 | 11.06 |
| 19 | 0.28 | 0.06 | 30.44 | 9.42 | 0.4 | 11.47 |
| 20 | 0.9 | 0.51 | 25.36 | 8.4 | 0.79 | 10.64 |
| 21 | 2.62 | 1.81 | 14.74 | 9.41 | 11.62 | 11.31 |
| 22 | 1.64 | 2.73 | 23.88 | 8.64 | 3.68 | 4.48 |
| 23 | 1.57 | 0.95 | 20.3 | 8.78 | 12.07 | 11.39 |
| 24 | 0.8 | 0.51 | 22.3 | 8.98 | 1.18 | 13.81 |
| 25 | 1.4 | 1.89 | 22.13 | 8.49 | 26.63 | 3.82 |
| 26 | 3.96 | 5.24 | 24.06 | 8.64 | 21.82 | 13.05 |
| 27 | 0.56 | 0.91 | 22.31 | 8.21 | 8.05 | 14.3 |
| 28 | 0.19 | 0.13 | 18.49 | 8.68 | 1.07 | 18.71 |
| 29 | 2.93 | 2.95 | 24.76 | 8.59 | 61.79 | 7.4 |
| 30 | 0.14 | 0.05 | 22.5 | 8.76 | 0.58 | 16.38 |
| 31 | 1.65 | 0.99 | 18.87 | 8.21 | 1.44 | 18.47 |
| 32 | 0.23 | 0.03 | 18.78 | 8.77 | 13.7 | 18.8 |
| 33 | 0.32 | 0.05 | 19.64 | 8.36 | 31.53 | 7.4 |
| 34 | 2.82 | 0.58 | 22.96 | 8.65 | 1.09 | 16.96 |
| 35 | 1.26 | 0.93 | 14.12 | 9.07 | 0.88 | 12.81 |
| 36 | 1.76 | 5.28 | 20.97 | 9.17 | 0.11 | 12.31 |
| 37 | 0.66 | 1.79 | 19.54 | 8.86 | 0.57 | 11.05 |
| 38 | 1.43 | 1.35 | 28.12 | 9.41 | 0.42 | 10.64 |
| 39 | 1.19 | 0.82 | 12.41 | 9.05 | 0.56 | 10.31 |
| 40 | 0.23 | 0.21 | 27.34 | 8.99 | 50.3 | 12.81 |
| 41 | 0.14 | 0.06 | 22.61 | 8.94 | 0.79 | 12.31 |
| 42 | 4.67 | 3.98 | 17.39 | 9.12 | 0.27 | 8.47 |

Note: STN (mg g^-1^), soil total nitrogen; STP (mg g^-1^), soil total phosphorus; EC (mS cm^-1^), soil electrical conductivity; K_soil_ (%), soil water stress coefficient.
